# Lactoferrin and Its Potential Impact for the Relief of Pain: A Preclinical Approach

**DOI:** 10.3390/ph14090868

**Published:** 2021-08-28

**Authors:** Beatriz Godínez-Chaparro, Fabiola Guzmán-Mejía, Maria Elisa Drago-Serrano

**Affiliations:** Departamento de Sistemas Biológicos, Universidad Autónoma Metropolitana Unidad Xochimilco, Calzada del Hueso No. 1100, Ciudad de México 04960, Mexico; bgodinez@correo.xoc.uam.mx (B.G.-C.); fabiolagm03@gmail.com (F.G.-M.)

**Keywords:** lactoferrin, pain, opioids, NO, NFκB

## Abstract

Pain is one of the most disabling symptoms of several clinical conditions. Neurobiologically, it is classified as nociceptive, inflammatory, neuropathic and dysfunctional. Opioids and nonsteroidal anti-inflammatory drugs (NSAIDs) are conventionally prescribed for the treatment of pain. Long-term administration of opioids results in the loss of analgesic efficacy, leading to increased dosage, tolerance, and addiction as the main drawbacks of their use, while the adverse effects of NSAIDs include gastric ulcer formation, intestinal bleeding, acute kidney injury, and hepatotoxicity. Lactoferrin is an iron-binding, anti-inflammatory glycoprotein that displays analgesic activities associated, in part, by interacting with the low-density lipoprotein receptor-related protein (LRP), which may result in the regulation of the DAMP–TRAF6–NFκB, NO–cGMP–ATP K^+^-sensitive channel and opioid receptor signaling pathways. This review summarizes and discusses for the first time the analgesic effects of lactoferrin and its presumable mechanisms based on pre-clinical trials. Given its anti-nociceptive and anti-inflammatory properties, lactoferrin may be used as an adjunct to enhance the efficacy and to decrease the tolerogenic effects of canonical therapeutic drugs prescribed for pain treatment.

## 1. Introduction

Pain is an unpleasant sensory and emotional experience with double-edged sword effects. On the one hand, pain acts as an alarm signal that warns us of potential damage and protects the body from injury, and on the other hand, pain can also be a needless sensation that covers up and worsens underlying damage, and, ultimately, pain can become a disease of its own [1]. Traditionally, pain is pathophysiologically classified as being either nociceptive or neuropathic. Nociceptive pain is defined as pain that arises from actual or threatened damage of non-neural tissue and is due to the activation of nociceptive neurons, also known as nociceptors (pain associated with active inflammation falls under this category) [2,3]. Neuropathic pain is pathological, arising as the consequence of damage to the peripheral nervous system or central nervous system; it is characterized by abnormal sensory processing known as allodynia (painful experience from a stimulus that does not normally trigger a pain response), and also includes hyperalgesia (an experience that increases pain from a stimulus that is normally perceived as painful) [4] and paresthesia (an abnormal sensation, whether spontaneous or evoked such as needle bites, tingling, itching, or even loss of sensitivity), among others [5]. In November 2017, the International Association for the Study of Pain (IASP) introduced a third mechanistic pain description in addition to nociceptive and neuropathic pain. The term nociplastic pain is defined as arising from the altered function of pain-related sensory pathways in the periphery and central nervous system, causing increased sensitivity [6]. Fibromyalgia, complex regional pain syndrome type 1, irritable bowel syndrome, headaches and orofacial pain, visceral pain syndromes, and musculoskeletal pain are all examples of pain conditions where nociplastic pain is typically present [7]. Nociplastic pain is typically described as dull, deep, and aching (adjectives classically associated with nociceptive pain), but with many patients describing a neuropathic quality (e.g., allodynia, hyperalgesia, and dysesthesia) [6].

The two families of drugs commonly used to mitigate pain are opioids and nonsteroidal anti-inflammatory drugs (NSAIDs), represented by their flagship drugs, morphine and aspirin, respectively. Historically, opioids have been the canonical and most frequent prototypical therapeutic agents prescribed for pain treatment in several clinical conditions [8]. Unfortunately, opioids induce undesirable side effects such as euphoria, sleepiness, respiratory depression, nausea, vomiting, itching, and constipation [8]. Moreover, after long-term opioid administration, opioid efficacy is markedly reduced, leading to tolerance, dependence, and ultimately drug abuse. Therefore, opioids are regarded as high risk and health-noxious painkiller drugs [8,9]. Although the consumption of opiates, in most cases, is the solution for inflammatory and nociceptive pain, various studies have shown that opioids are not effective in treating pathological pain such as neuropathic and functional pain [10].

Nonsteroidal anti-inflammatory drugs have anti-inflammatory, anti-pyretic, and analgesic properties [11]. NSAIDs have been the conventional drugs of choice for the treatment of pain, particularly pain that has a strong inflammatory and nociceptive component [10]. Unfortunately, NSAIDs also causes adverse effects, including gastric ulcer formation, intestinal bleeding, acute kidney injury, and hepatotoxicity [12].

For a long time, neuropathic and nociplastic pain had been considered intractable because the use of common analgesics such as NSAIDs and opioids does not relieve these types of pain. Currently, the first line of treatment for neuropathic pain is based on the use of antidepressant drugs such as amitriptyline and duloxetine, as well as anticonvulsant drugs such as gabapentin and pregabalin [13]. The latter are widely used to treat neuropathic pain as well as fibromyalgia and other functional pain states. Antidepressant and anticonvulsant drugs have analgesic and anti-hyperalgesic effects. They are not pain relievers in a classical sense, but they help to reduce discomfort and the unpleasant emotional component of neuropathic and nociplastic pain [10]. Non-opioid analgesics such as tricyclic antidepressants, and gabapentinoids that act on the central nervous system, can provide some benefit. Unfortunately, these drugs cause various adverse reactions such as dizziness, drowsiness, confusion, lethargy, and problems walking or performing activities that require dexterity [14], so many patients discontinue treatment.

Several drugs have been proposed as adjuncts for traditional analgesic drugs in order to increase their efficacy and to reduce their noxious side effects [15]. In this regard, milk is a source of natural components with anti-nociceptive and anti-inflammatory properties, including bovine lactoferrin (bLf) [16,17]. Bovine lactoferrin is generally recognized as a safe substance (GRAS) by the Food and Drug Administration (FDA, USA) [18]. In animal studies, the oral administration of bLf has not shown any obvious toxic effects with doses up to 2 g/kg body weight once daily for 13 weeks [19]. In addition, clinical studies have shown that the oral administration of up to 7.2 g of bLf each day for 8 weeks [20], as well as the administration of bLf or human hLf (100 mg twice daily for 1 month) tablets, is well tolerated; no serious adverse events were observed during the treatment of adults [21,22]. Moreover, when bLf was orally administrated to neonates (100 mg each day for 6 weeks or 850 mg/L/12 months), no adverse effects or intolerance to treatment occurred [23,24]. Currently, the therapeutic dosage of lactoferrin(Lf) is yet to be standardized; different dosages have been tested in several trials. Nonetheless, Lf is a safe product for human use. In fact, there is growing evidence supporting the therapeutic application of purified Lf to several clinical conditions, either as a milk supplement or an additive in pharmaceutical products [25,26,27,28].

Bovine lactoferrin is a multifunctional, iron-binding glycoprotein with anti-inflammatory effects produced by reducing the activation of neural inflammatory signaling pathways associated with pain [29]. Taking into account their effects against pain, potential therapeutic and prophylactic applications of bLf and bLf-peptide derivatives to several pathologic conditions—for example, osteoarthritis—have been proposed [30]. Thus, this manuscript is focused on reviewing the potential anti-nociceptive properties of bLf, as well as its mechanisms of action, as documented in animal models of experimentation. This contribution has been proposed in order to consider the application of bLf as an adjunct to opioids and other painkiller drugs to potentiate their efficacy and help reduce the risk of tolerance, dependence, addiction, and life-threatening drug abuse.

## 2. Lactoferrin Overview

Lactoferrin is an 80 kDa iron-binding globular glycoprotein from mammals that is abundantly secreted in milk and colostrum. Cows and humans are regarded as the major producers of Lf among mammals by a wide margin [31]. Functionally and structurally, bLf and human lactoferrin (hLf) are the most characterized mammalian Lfs. The canonical Lf structure consists of a single polypeptide chain folded into homologous N- and C-terminal lobes linked covalently by a three-turn α-helix. Each N- and C-terminal lobe contains two equal subdomains, N1-N2 and C1-C2; each forms a deep cleft, where one ferric ion (Fe^3+^) is tightly bound in synergistic cooperation with a bicarbonate ion (HCO_3_^−1^) [32]. The ability of Lf to bind Fe^3+^ reversibly generates a full diferric-loaded Lf known as holoLf and a diferric-depleted Lf termed apoLf [33].

The enzymatic cleavage of Lf by pepsin at the N-terminal domain renders a peptide derivative known as lactoferricin (Lfcin), which lacks metal-binding activity [34]. Human lactoferricin (hLfcin) is a peptide fragment corresponding to N1–47 of full-length Lf, with two subunits, namely fragments 1–11 and 12–47, linked by a disulfide bridge between the residues Cys20 and Cys37. Bovine lactoferricin (bLfcin) consists of a 25-residue sequence corresponding to N17–41 of bLf connected by a disulfide bridge between the residues Cys19 and Cys36 [34].

Lactoferrin is capable of carrying out some modulatory actions due to its interaction with multiple receptors expressed on a wide array of target cells. Receptors that bind to Lf include intelectin-1 (omentin-1), CD14, chemokine receptor 4 (CXCR4), and low-density lipoprotein receptor-related protein (LRP), among many others [33,34,35]. Intelectin-1 is expressed in the small intestine and enables Lf uptake, that is, the binding and endocytosis of Lf in a calcium-dependent manner. After internalization, Lf is able to trigger the extracellular signal-regulated kinase (ERK)1/2 signaling pathway. CD14 is a lipopolysaccharide (LPS) coreceptor that enables the interaction between LPS, a glycolipid produced by gram-negative bacteria, and Toll-like receptor 4 (TLR4), a component of the innate immune response that can also bind to host-derived endogenous ligands [36]. The outcome of bLf on the LPS–CD14 interaction is discussed below [37,38]. CXCR4 is a potent chemokine for the receptor-mediated recruitment of leukocytes. bLf interaction with CXCR4 induces the activation of the AKT pathway, as demonstrated in HaCaT human keratinocytes and Caco-2 human intestinal cells [39].

Low-density lipoprotein receptor-related protein is a transmembrane glycoprotein widely expressed in the brain (central nervous system), hepatocytes, smooth muscle cells, and fibroblasts. It consists of an extracellular 85-kDa light chain and a 515-kDa heavy chain that spans the cell membrane. LRP1 is the most multifunctional member of the LDL receptor family. LRP1 has been implicated in two main biological functions: the uptake of numerous ligands and the regulation of cell signaling pathways. It is thought that bLf transcellular transport across the brain endothelium to enter the cerebrospinal fluid takes place through the blood-brain barrier (BBB) via LRP receptor–mediated transcytosis [40]. The BBB-bLf endocytosis via LPR is a presumable mechanism through which Lf exerts modulatory actions on the central nervous system. In addition, binding of Lf to LRP1 induces the activation of the ERK1/2 signaling pathway in osteoblasts, fibroblasts, keratinocytes, adipocytes, and neurons [39,41].

Multiple activities of Lf on the nervous system have been demonstrated via several experimental and in vitro assays. Among the pharmacological properties is the ability of bLf to blunt the impact of stress-associated neuroendocrine components [42]. Lf functionally encompasses the regulation of players with a pivotal role in inflammation and the maintenance of anti-oxidative balance. In this regard, bLf modulates pro-inflammatory cytokine generation [43], iron absorption [44], and the production of reactive oxide species (ROS) and reactive nitrogen species (RNS) [45,46]. These properties seem to be intimately intermingled with the anti-nociceptive and anti-inflammatory effects of bLf [16,29,47], which will be described in more detail in the following section.

### Lactoferrin: Modulatory Properties on the Inflammatory Response

Inflammatory responses can be triggered by both host-derived molecules—namely damage-associated molecular patterns (DAMPs), which occur following cell damage—and by microbial-derived products—namely pathogen-associated molecular patterns (PAMPs), including flagellin, toxins, peptidoglycan, LPS, etc. [48]. The recognition of PAMPS or DAMPS occurs by innate host receptors termed pattern recognition receptors (PRRs) present in the plasma membrane and endosomes. After cognate ligand–receptor interaction takes place, intracellular signaling pathways are triggered and culminate in the activation of nuclear factor κB (NFκB) and AP1 transcription factors [36]. Both modulate the expression of genes encoding proteins that synthesize pro-inflammatory cytokines and chemokines. In addition, the inflammatory response encompasses secondary pro-inflammatory mediators such as prostaglandin E2 (PGE2), produced from cyclooxygenase (COX), and nitric oxide (NO), generated by nitric oxide synthase (NOS) [49,50].

TLR4 expressed on the cell surface has been found to bind to not just LPS, but also DAMPs including heat shock proteins (HSPs), high mobility group box protein (HMGB1), oxidized low density lipoprotein (LDL), and fibronectin, among others [36]. It is worth underlining that at present, the role of Lf on the outcome of DAMP–TLR4 ligation is unknown; however, a growing number of studies have evidenced the modulatory actions of Lf on the LPS–TLR4 interaction. As described in endothelial cells, LPS is associated with soluble CD14 (sCD14), and the LPS–sCD14 complex acts properly as a ligand for TLR4 [37]. As demonstrated in mononuclear cells, LPS interacts with LPS-binding protein (LBP), which enables the ligation of LPS with membrane-associated CD14 (mCD14) to generate the LPS–mCD14 complex. The LPS tethered to mCD14 is transferred to MD2, and the LPS–MD2 protein complex acts as the actual ligand for TLR4. After the “catch and pass” LPS detour ends, the LPS–TLR4 interaction induces an inflammatory response that may potentially cause chronic inflammatory disorders if excessive stimulation occurs [37]. This evidence may support the anti-nociceptive action of Lf by dampening the DAMP–TLR4 pathway activation, as proposed in this review.

In vitro studies in epithelial cells, macrophages, and monocytes, it has been observed that LPS increases the expression of pro-inflammatory cytokines such as interleukin 1β (IL1β), IL6, and IL8 via the activation of NFκB. The response of these pro-inflammatory markers triggered by LPS was blunted by Lf pre-treatment [51,52,53]. In line with these findings, in mice treated systemically with LPS to induce endotoxemia, Lf decreased the plasma concentrations of PGE2, NO, and tumor necrosis factor α (TNFα) [46,54]. Moreover, Lf modulates inflammation by blocking the binding of LPS to sCD14, mCD14, and LBP. This disturbs the formation of the LPS–sCD14 or LPS–LBP–mCD14 complexes and thus attenuates the TLR4 signaling pathway [35]. On the other hand, Lf, by itself, is capable of triggering a pro-inflammatory response via a TLR4-independent signaling pathway [55]. Lf may also inhibit the LPS-induced expression of adhesion molecules, such as E-selectin and Intercellular Adhesion Molecule 1 (ICAM-1), resulting from the activation of endothelial cells associated with microvascular thrombosis, with a fatal outcome [56]. Furthermore, an in vitro model using chondrocytes demonstrated that Lf inhibits the IL1β-induced activation and nuclear translocation of NFκB and messenger RNA (mRNA), the protein expression of COX-2, and the production of PGE2 [49]. Taken together, these data suggest that Lf can inhibit the TLR4–NFκB pathway and COX activation; these actions probably account for the anti-nociceptive effects.

## 3. Lactoferrin: Animal Models to Study Anti-Nociceptive Properties

### 3.1. Anti-Nociceptive Effect Induced by Lactoferrin in Animal Models

Although nociception and pain have sometimes been treated as synonyms, they represent utterly divergent conditions. Nociception refers to the neurophysiologic manifestations generated by a noxious stimulus, while pain involves the perception of an aversive stimulus, which requires the capacity of abstraction and the elaboration of sensory impulses [57]. Pain does not just involve nociception and nocifensive withdrawal, but also encompasses the negative affective component of pain [58]. Pre-clinical studies have sought to reduce nociception through the evaluation of new bioactive molecules with potential analgesic activity, such as Lf, and understanding the mechanisms that underlie the development and maintenance of pain. In this sense, several animal models have been adopted for nociception research, and their use has yielded advances in our knowledge of the neurobiology of pain and nociception.

The impact of Lf over nociceptive pain has been documented in experimental models. These include (i) the hot plate test that evaluates the thermal (54 ± 0.5 °C) nociception reflex; (ii) acetic acid-induced abdominal constriction, a model of visceral nociceptive pain; (iii) the formalin test, in which the injection of formalin into the skin of rodent hind paws causes a spontaneous pain-related flinch, resulting in a nociceptive response that can be divided into two phases; and (iv) adjuvant-induced arthritis, which is a model of chronic immune-mediated joint inflammation induced by injection of Complete Freund’s Adjuvant (CFA).

In rats, a single intraperitoneal administration of bLf (100 mg/kg) reduced formalin-induced nociceptive behavior during both phases of the test [59]. Moreover, the injection of bLf (100 mg/kg, intraperitoneal (i.p.)) was able to inhibit thermal nociception and visceral nociception in the hot plate and acetic acid writhing tests, respectively [16]. Interestingly, animals fed with bLf (1 g/kg each day) for 4 weeks showed a reduction in formalin-induced flinching behavior in both phases [16]. In addition, a single oral administration of bLf (100 mg/kg) to rats showed an anti-hyperalgesic effect that lasted 6 days after its administration for CFA-induced arthritis [60]. Taken together, these data suggest that bLf administered by systemic and oral routes is able to produce an anti-nociceptive effect. Experimental findings have shown that bLf was present in the cerebrospinal fluid of piglets [61] and calves after oral administration. Additionally, Lf and the LRP have been identified in the CNS, especially in the brain [40]. Thus, orally administered bLf could act in the CNS and induce anti-nociception. In concordance with these data, spinal injections of bLf (1.25–1250 pmol/rat) decreased formalin-induced nociceptive behavior during phases 1 and 2 [59].

On the other hand, peripheral administration by injection of bLf (3.85 nmol/paw) and hLf (1.25 nmol/paw) decreased the formalin-induced nociceptive behavior during phases 1 and 2 in rats [62]. These data suggest that bLf and hLf could induce peripheral anti-nociceptive activity. Several studies have demonstrated that Lf receptors such as LRP are present in the brain [63,64,65]. Unlike the peripheral nervous system, the presence of LRP has been observed in various types of peripheral tissues, including the liver, intestines, heart, salivary gland, and pancreas [66]. Given that Lf performs marked inhibitory activity in the peripheral nervous system, it is likely that the Lf receptor may exist in the peripheral nerves. Additional studies are required to clarify this point. Table 1 summarizes the effects of Lf on nociceptive pain in animal models.

### 3.2. Anti-Allodynic and Anti-Hyperalgesic Effects Induced by Lactoferrin in Animal Models

Anti-allodynic and anti-hyperalgesic effects of Lf have been documented in experimental models of neuropathic pain. These include (i) a chronic constriction injury model (CCI), in which the sciatic nerve is exposed and isolated with four loose ligatures placed around it [67]; (ii) a mental nerve transection (MNT) model, in which the mental nerve (the third branch of the trigeminal nerve) is exposed and ligated, then a section distal to the ligation and distal nerve stump is resected [68]; (iii) a herniation model, in which the L5 nerve root and dorsal root ganglion are exposed, then an L-shaped stainless steel rod is inserted toward the intervertebral foramen to compress the L5 nerve root [69]; and (iv) an oxaliplatin mouse model, which involves a single intraperitoneal injection of oxaliplatin to induce neurotoxicity caused by platinum accumulation [70,71]. In all these models of neuropathic pain, the lesion of the central or peripheral nervous system is able to develop allodynia and hyperalgesia.

Experimental findings in rats have demonstrated that a single administration of bLf (1, 10, or 100 µg/rat, intrathecal (i.t.)) reduces thermal hyperalgesia induced by CCI; the time to reach the maximal anti-hyperalgesic effect was 30 min after and receded gradually over approximately 180 min [72]. Moreover, bLf (200 µg/rat, i.t.) increased the paw withdrawal threshold in animals that had undergone MNT surgery. The anti-allodynic effect induced by bLf was evident at 24 h and 48 h after administration [29], suggesting that bLf alleviates extra-territorial allodynia.

In another study, a single intraperitoneal injection of bLf (100 mg/kg) exerted an anti-allodynic effect in rats that had undergone herniation [69]. By contrast, a single bLf dose (100 or 200 mg/kg, i.p.) did not show differences at either dose for mechanical hyperalgesia and tactile allodynia induced by CCI [47]. However, sub-acute administration of bLf (50, 100, 200 mg/kg each day for 15 days, i.p.) reverted the mechanical hyperalgesia and tactile allodynia in neuropathic rats [47].

Daily intraperitoneal administration in rats for 2 weeks with recombinant human lactoferrin (rhLf) or hLf-IgG Fc fusion protein decreased the allodynia induced by oxaliplatin. The hLf-IgG Fc fusion protein is a biotechnological product for improving the blood stability of pharmaceutical proteins [73]. These data suggest that the route of administration and the animal model conducted to test neuropathic pain influence the anti-allodynic and anti-hyperalgesic effects produced by bLf or rhLf. These compounds enter the central nervous system through the BBB via receptor-mediated transcytosis [40]. Therefore, it is expected that bLf bioavailabilty at the spinal level takes time to impact the anti-nociceptive activity. Table 2 summarizes the Lf anti-nociceptive activities in experimental models of neuropathic pain.

## 4. Mechanisms of Action Underlying the Anti-Nociceptive Activity of Lactoferrin

### 4.1. Role of the TRAF6–NFκB Signaling Pathway

Several studies have demonstrated that the activation of NFκB takes place in dorsal root ganglia and the spinal cord, both of which are involved in the transmission and processing of pain. Consistently, an increase in NFκB activation has been observed in lumbar dorsal root ganglia following partial CCI [74], and in the spinal cord after CFA-induced paw inflammation [75]. Moreover, a single endoneural injection of NFκB decoy at the site of the nerve lesion reverted thermal hyperalgesia and suppressed the expression of the inflammatory cytokines TNFα, IL1β, and IL6, as well as inducible nitric oxide synthase (iNOS) and interferon γ (IFNγ) [76]. Intrathecal pre-treatment with NFκB decoy reduced mechanical allodynia and thermal hyperalgesia, and suppressed the activation of NFκB and the expression of COX-2 following unilateral hind paw inflammation induced by CFA [77]. In line with these results, in vitro studies have demonstrated that Lf, bLf, or LFP-20 (a porcine Lf peptide) suppressed phosphorylated (p)-p38 mitogen-activated protein kinase (MAPK), phosphorylated (p)-p65 subunit of NFκB, and inflammatory cytokines such as IL6 and IL1β [78,79,80]. As found in neuropathic rats, TNFα increases were significantly lower with bLf (200 mg/kg/ daily for 15 days than the treatment with bLf (50 or 100 mg/kg/ daily for 15 days) and saline [47].

**Figure 1:**
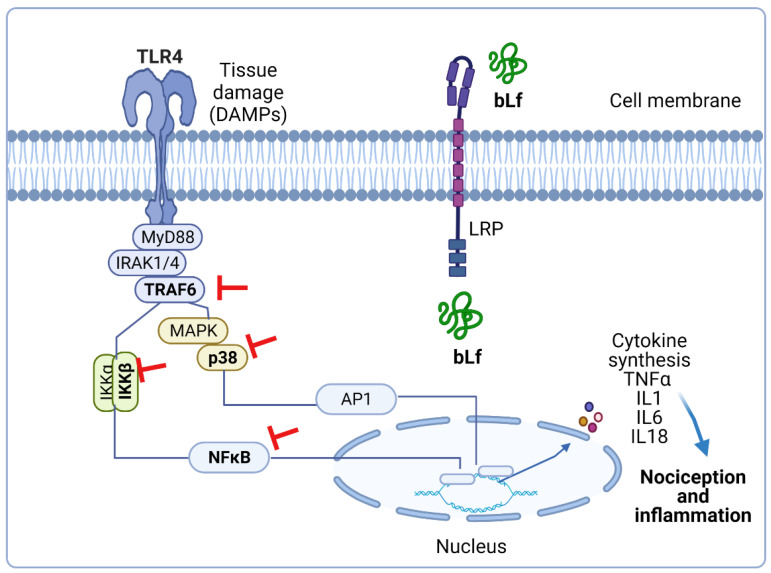
Presumable effect of bovine lactoferrin (bLf) on the damage-associated molecular pattern (DAMP)–Toll-like receptor 4 (TLR4)–TRAF6–nuclear factor κB (NFκB) signaling pathway. Tissue damage leads to host-derived endogenous DAMPs. The latter activate MyD88-dependent pathway via TLR4 signaling, thus initiating the transcription of cytokines that produce nociception and inflammation. Pre-treatment with bLf prevents inflammation by avoiding the activation of TRAF6, IKK, p38, and p65. This figure was created with Biorender.com (accessed on 25 August 2021).

In addition, daily administration of bLf (100 mg/kg, per oral) for 18 days suppressed TNFα and IL1β production and increased IL10 production in the LPS-stimulated adjuvant arthritis rats [60]. Furthermore, intrathecal administration of bLf prevented the increased expression of p-p38, p-p65, p-IKKβ, and IL18 in the trigeminal subnucleus caudalis in neuropathic rats [29]. These data suggest that immunomodulatory properties of bLf that lead to downregulation of p-p38, p-p65, p-IKKβ, IL18, TNFα, and IL1β and upregulation of IL-10 could be beneficial in the treatment of inflammatory and neuropathic pain. In this context, Horie et al. [29] demonstrated that after MNT, p-38 was present in microglia and p-65 was present in astrocytes. In vitro studies have suggested that TRAF6 and bLf are associated with each other in the cytoplasm [79]. From this perspective, intrathecal administration of bLf is subsequently endocytosed via LRP1 to bind to endogenous TRAF6 [29]. Due to binding to TRAF6, bLf decreased the expression of p-p38 in microglia; p-p65 in astrocytes; and p-38, p-p65, p-IKKβ, IL18, and TNFα in the brains of neuropathic rats [29]. According to these findings, and assuming that tissue damage drives the release of DAMPs as ligands of TLR4, Figure 1 represents the presumable role of bLf on the DAMP–TLR4–TRAF6–NFκB signaling pathway that ameliorates the inflammatory response associated with nociceptive responses.

### 4.2. Role of the NO–cGMP–ATP-Sensitive K^+^ Channel Signaling Pathway

NO is a signaling molecule that plays an important role in acute and chronic nociception states at the peripheral and central levels [81]. The anti-nociceptive effect of NO is accompanied by the activation of the neuronal nitric oxide synthase (nNOS), which induces the formation of cyclic guanosine monophosphate (cGMP) via guanylate cyclase. The NO–cGMP signaling pathway is present in neurons in the spinal cord and has been implicated in synaptic plasticity, such as central sensitization [82]. The activation of cGMP-dependent protein kinase (PKG) phosphorylates and opens ATP-sensitive K^+^ channels. Activation of ATP-sensitive K^+^ channels promotes neuron hyperpolarization through an increase in intracellular K^+^ influx, and therefore relieves nociception and reestablishes the normal threshold of nociceptive neurons [81,83].

Several studies have demonstrated that the NO–cGMP–ATP-sensitive K^+^ channel pathway induces analgesia and modulates the anti-nociceptive effect of analgesics such as NSAIDs, natural products, antiepileptics, and opioids [81,84,85,86]. In this sense, intrathecal injection of L-NAME (a nonselective NOS inhibitor) or 7-NI (an nNOS-specific inhibitor) reverted the anti-allodynic effect of bLf, also administered intrathecally, in neuropathic rats. Moreover, both inhibitors reverted the anti-nociceptive effect on the formalin-induced behavior in both phases induced by intrathecal injection of bLf [59,72]. In addition, peripheral coadministration of L-NAME with bLf reverted bLf-induced anti-nociception in both phases in the formalin test [59]. The data suggested that anti-nociceptive effects at peripheral or spinal levels were mediated by bLf-induced NO production. However, it has been reported that chronic intraperitoneal administration of bLf reverts the increased expression of iNOS and nNOS in the lumbar spinal cord of neuropathic rats [47]. It is worth reinforcing that NO has a dual role at the spinal or peripheral level in the regulation of pain processes: it can mediate nociception or induce an anti-nociceptive effect [81]. Controversial data about the anti-hyperalgesic or anti-allodynic bLf effects associated with an increase or a decrease in NO production may result from differences in dosage, time, and route of bLf administration in neuropathic rats [47,72]; however, the bimodal impact of NO on neuropathic pain by modulating the activation of nociceptive receptors might be involved. Indeed, NO triggers the activation of neuronal nociceptive receptors, but neurons with greater excitability are prone to be inhibited by NO, and this inhibitory action is mediated by cGMP [87].

**Figure 2:**
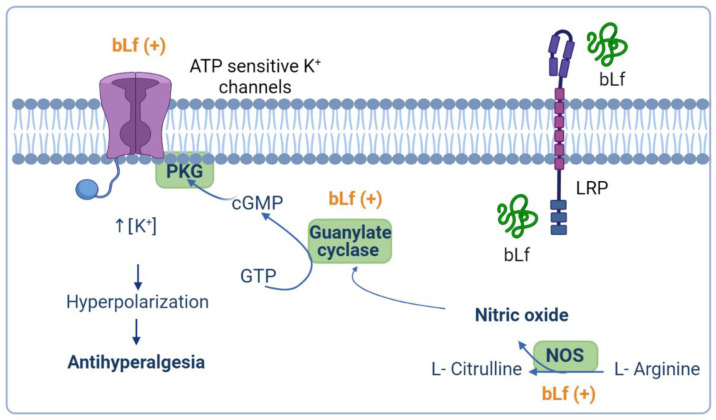
Presumable anti-hyperalgesic activity of bovine lactoferrin (bLf) through the nitric oxide (NO)–cyclic guanine monophosphate (cGMP)–ATP-sensitive K^+^ channel signaling pathway. NO has an anti-nociceptive effect based on the formation of cGMP via guanylate cyclase activation. Production of cGMP leads to cGMP-dependent protein kinase (PKG) phosphorylation and the concomitant opening of ATP-sensitive K^+^ channels. Activation of ATP-sensitive K^+^ channels increases the intracellular K^+^ levels that, in turn, drive hyperpolarization of the neuron, and therefore relieves nociception and reestablishes the basal threshold of the neuron. bLf may exert its anti-hyperalgesic effect by activating the NO–cGMP–ATP- sensitive K^+^ channel signaling pathway. This figure was created with Biorender.com.

Besides, intrathecal administration of guanylate-cyclase inhibitors such as 1H-1,2,4-oxadiazolo4,3-aquinoxalin-1-one (ODQ) or KT-5823 blocked the anti-hyperalgesic effect induced by bLf in rats subjected to CCI [72]. Therefore, these findings suggest that the anti-hyperalgesic effects of bLf are mediated by the induction of NO production with the subsequent activation of guanylate cyclase. Moreover, pre-treatment with glibenclamide (an ATP-sensitive K^+^ channel blocker) reverted the bLf anti-hyperalgesic effects, suggesting a role for ATP-sensitive K^+^ channels in bLf induced anti-hyperalgesia [72]. Taken together, these studies suggest that the anti-hyperalgesic effect induced by bLf is mediated by the NO–cGMP–ATP-sensitive K^+^ channel pathway in rat models of neuropathic pain (Figure 2).

### 4.3. Role of the Opioidergic System

Opioid analgesics are widely used in the management of pain. Opioids produce analgesia through their actions in the central nervous system [88], but they also relieve peripheral hyperalgesia in conditions involving inflammation or prolonged nociceptive stimulation [89] via activation of the µ, κ, and δ opioid receptors that are present in nociceptor endings located peripherally [90,91]. Several studies have demonstrated that the anti-nociceptive effect induced by Lf is modulated by the opioid system. Hayashida et al. [16] demonstrated that the anti-nociceptive effect induced by bLf (100 mg/kg, i.p.) is reverted by the intraperitoneal injection of naloxone (a non-selective opioid receptor antagonist) in nociceptive behavior induced by formalin and hot plate tests. Moreover, subcutaneous administration of naloxone (100 mg/kg, p.o.) reverted the anti-hyperalgesic effect induced by bLF in rats treated with CFA [60]. In addition, the anti-nociceptive effect of bLf (10 µg/rat, i.t.) was prevented by the intrathecal injection of naloxone or CTOP (a selective μ opioid receptor antagonist) but not of norBNI (a selective μ opioid receptor antagonist) during both phases of the formalin test [16]. Moreover, the coadministration of naloxone (10 µg/rat, i.t) or CTOP (1 µg/rat, i.t) reverted the bLf-induced anti-nociceptive effect in both phases of the formalin test [59]. Furthermore, the peripheral coadministration of naloxone (20 µg/paw) or CTOP (2 µg/paw) with bLf (1.28 nmol/paw) reverted the bLf-induced anti-nociception in both phases in the formalin test [62]. Taken together, these studies suggest that the anti-nociceptive effect of bLf is mediated, at least in part, by the opioidergic system through the activation of µ opioid receptors at the central and peripheral levels in different nociceptive pain models.

Regarding neuropathic pain, the intraperitoneal administration of naloxone, CTOP, or norBNI was able to prevent the anti-allodynic effect induced by bLf (100 mg/kg/ daily for 15 days in rats that had undergone CCI [47]. By contrast, the intrathecal administration of naloxone and CTOP did not prevent the anti-hyperalgesic effect induced by a single intrathecal injection of bLf [72]. These data suggest that sub-acute but not acute administration of bLf induces anti-allodynic effects that are mediated by the opioidergic system through the activation of µ, κ, and γ opioid receptors at the systemic level.

### 4.4. Potentiation of Peripheral and Spinal µ Opioid Receptor-Mediated Anti-Nociception by Bovine Lactoferrin

**Figure 3:**
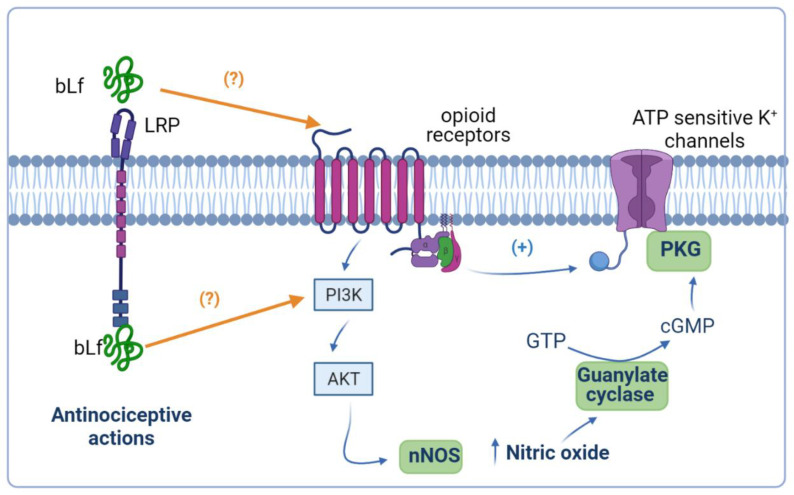
Anti-nociceptive potentiation of bovine lactoferrin (bLf) and opioids. Ligands of opioid receptors activate the phosphoinositide 3-kinase (PI3K)–AKT signaling pathway, favoring the production of nitric oxide (NO). The generation of NO culminates in the activation of the NO–cyclic guanine monophosphate (cGMP) signaling pathway that, together with opioid receptor-mediated protein G-activation, opens the ATP-sensitive K^+^ channels. It is probable that bLf potentiates the effects of opioids because it can act on opioid receptors and/or induce the activation of the PI3K–AKT signaling pathway. This figure was created with Biorender.com.

Previous evidence has demonstrated that peripheral opioid agonist-induced anti-nociception is mediated by the NO–cGMP pathway [92,93]. In this context, bLf-induced peripheral anti-nociception was reverted by a NOS inhibitor, and a subeffective dose of bLf potentiated the morphine-induced anti-nociceptive effect, which was also reverted by a NOS inhibitor [62]. On the other hand, a subeffective dose of bLf (1.025 nmol/h, i.t.) potentiated the µ opioid agonist (morphine, 27 nmol/h, i.t.)-induced anti-nociceptive effect in the tail-flick test [59]. Moreover, spinal injection of bLf increased the µ opioid receptor system through NO production and induced anti-nociception without tolerance [59]. These data suggest that bLf acts as an enhancer of the spinal opioidergic system via an NO-mediated mechanism. Interestingly, Cunha et al. [94] described that the activation of the NO pathway by morphine depended on an initial stimulation of the phosphoinositide 3-kinase (PI3K)–AKT signaling pathway, which in turn might cause the stimulation of nNOS to enhance NO production. Consequently, NO, through the stimulation of cGMP/PKG, causes the upregulation of K^+^ currents of ATP-sensitive K^+^ channels and promotes the hyperpolarization of primary nociceptive neurons [94]. Hence, a subeffective dose of bLf (300 mg/kg, p.o.) induced potentiation of morphine-induced anti-nociception [95]. Moreover, the intraperitoneal administration of 7-NI blocked the potentiated anti-nociceptive effect by coadministration of morphine and bLf [95]. Furthermore, Hayashida et al. [62] reported that the peripheral administration of bLf also potentiated the morphine-induced anti-nociceptive action. There has been no evidence that Lf can activate the PI3K–AKT signaling pathway in animal models of pain. In vitro studies have suggested that Lf downregulates thromboxane 2 (TXA2) and upregulates prostacyclin 2 (PGI2) through activating the PI3K–AKT–ERK1/2 signaling pathway in human umbilical vein endothelial cells [96]. Moreover, Lf induced the activation of AKT in human breast adenocarcinoma (MCF) cells; however, the treatment of MCF cells with a PI3K inhibitor almost completely blocked Lf-stimulated cell cycle progression [97]. Taken together, these data suggest that this peripheral potentiation of anti-nociceptive activity of bLf with morphine involves the NO and PI3K–AKT signaling pathways (Figure 3). However, further investigation in the future is necessary to clarify this point.

## 5. Conclusions

Experimental rat models of nociceptive and neuropathic pain have provided substantive evidence regarding the potential analgesic properties of bLf. Currently, several drugs have been proposed as coadjutants for traditional pain therapies in order to increase their efficacy and reduce their noxious side effects [15]. bLf has anti-nociceptive and anti-inflammatory properties of presumable use as an adjunct of drugs for pain treatment for several conditions, including osteoarthritis and neuropathic pain [30,79]. This review provides tentative mechanisms that support the potential application of bLf as a coadjutant for opioid and NSAID therapy, and other painkillers such as anti-depressive or anti-convulsive drugs, to potentiate their efficacy and to help reduce the side effects and risks of tolerance, dependence, addiction, and life-threatening drug abuse. Little is known about the mechanisms of action involved in the anti-nociceptive effect induced by Lf. So far, the possible bLf-mediated mechanisms include its involvement in the DAMP–TRAF6–NFκB and NO–cGMP–K^+^-ATP sensitive channel signaling pathways, and by the opioidergic system through the activation of µ, κ, and δ opioid receptors. However, future studies that ascertain the mechanism of bLf are required to gain substantive insights about its efficiency as an adjunctive and its potential hazard effects.

The application of lactoferrin at the human scale remains distant given that most of the knowledge about its properties has come from animal models. However, Lf is a safe product with no side effects observed from clinical trials that may be included as an adjunct of opioids and other painkiller drugs prescribed to humans to potentiate their efficacy and to help reduce the side effects and risks of tolerance, dependence, addiction, and life-threatening drug abuse.

## Figures and Tables

**Table 1:** Effects induced by lactoferrin in animal models of nociceptive pain.

Nociceptive Pain		
Animal Model	Lactoferrin Treatment	Effect
Formalin test	bLf (30–300 mg/kg, i.p.) bLf (1 g/kg each day for 4 weeks, i.p.) bLf (100 µg/rat, i.t.) bLf (0.385–3.85 nmol/paw, i.p.) rhLf (1.25 nmol/paw, i.p.)	Anti-nociception in phases 1 and 2 [16,59,62]
Hot plate test	bLf (100 mg/kg, i.p.)	Thermal anti-nociception [16]
Acetic acid writhing test	bLf (1 or 3 mg/kg, i.p.)	Visceral anti-nociception [16]
CFA	bLf (100 mg/kg, p.o.)	Anti-inflammatory and anti-hyperalgesia [60]

Abbreviations: CFA—Complete Freund’s Adjuvant; i.p.—intraperitoneal; i.t.—intrathecal; p.o.—per os (oral); bLf—bovine lactoferrin; rhLf—recombinant human lactoferrin.

**Table 2:** Effects induced by lactoferrin in animal models of neuropathic pain.

Neuropathic Pain		
Animal Model	Lactoferrin Treatment	Effect
CCI	bLf (100 µg/rat, i.t.)	Thermal anti-hyperalgesia [72]
	bLf (100 and 200 mg/kg/ daily for 15 days, i.p.)	Mechanical and thermal anti-hyperalgesia Tactile anti-allodynia [47]
MNT	bLf (200 µg/rat, i.t.)	Tactile anti-allodynia Mechanical anti-hyperalgesia [29]
Herniation	bLf (100 mg/kg, i.p.)	Anti-allodynia [69]
Oxaliplatin	rhLf (100 mg/kg, i.p., 3 doses (once per week for 3 weeks)); rhLf-Fc (100 mg/kg, i.p., 3 doses (once per week for 3 weeks); continuous infusion of rhLf or rhLf-IgGFc (10 g/kg/ at days 0, 4, 7, 11, 14, 17, 21, 24 and 28)	Mechanical anti-allodynia [73]

Abbreviations: bLf—bovine lactoferrin; CCI—chronic constriction nerve injury; i.p.—intraperitoneal; i.t.—intrathecal; MNT—mental nerve transection; p.o. per oral; rhLf—recombinant human lactoferrin; rhLf-IgG-Fc—recombinant human lactoferrin; IgG-Fc—fusion protein.

## Data Availability

Data sharing not applicable.
